# Towards a general theory of implementation

**DOI:** 10.1186/1748-5908-8-18

**Published:** 2013-02-13

**Authors:** Carl May

**Affiliations:** 1 Faculty of Health Sciences, University of Southampton, Building 67 (Nightingale), University Road, Highfield, Southampton SO17 1BJ, UK

## Abstract

Understanding and evaluating the implementation of complex interventions in practice is an important problem for healthcare managers and policy makers, and for patients and others who must operationalize them beyond formal clinical settings. It has been argued that this work should be founded on theory that provides a foundation for understanding, designing, predicting, and evaluating dynamic implementation processes. This paper sets out core constituents of a general theory of implementation, building on Normalization Process Theory and linking it to key constructs from recent work in sociology and psychology. These are informed by ideas about agency and its expression within social systems and fields, social and cognitive mechanisms, and collective action. This approach unites a number of contending perspectives in a way that makes possible a more comprehensive explanation of the implementation and embedding of new ways of thinking, enacting and organizing practice.

## Background

*That we are never alone in carrying out a course of action requires but a few examples.* Bruno Latour [1].

Understanding and evaluating the implementation of healthcare interventions in practice is an important problem for healthcare managers and policy-makers [2], and also increasingly for patients and others who must operationalize them beyond the boundaries of formal clinical settings [3,4]. For the research community, applied research in this domain forms a focus for the new interdisciplinary field of ‘Implementation Science’ [5], and the development of implementation theory [6,7] that provides a foundation for understanding, designing, predicting, and evaluating dynamic implementation processes. Implementation Science, like other closely related fields (for example, Health Services Research, Health Technology Assessment, and Improvement Science), needs comprehensive, robust, and rigorous theories that explain the social processes that lead from inception to practice.

This paper is intended to make a contribution to implementation theory. It does so by linking an existing theory – Normalization Process Theory [8-10], which characterizes implementation as a social process of collective action – with constructs from relevant sociological theories of social systems and fields, and from relevant social cognitive theories in psychology. The general approach here is to integrate these to provide a more comprehensive explanation of the constituents of implementation processes. This takes the form of a theoretical framework that characterizes and explains implementation processes as interactions between ‘emergent expressions of agency’ *(i.e*., the things that people do to make something happen, and the ways that they work with different components of a complex intervention to do so); and as ‘dynamic elements of context’ (the social-structural and social-cognitive resources that people draw on to realize that agency). The objective of this integrative approach to theory is to set out some of the core elements of a general theory of implementation. The theory presented is one that emphasizes agentic contributions and capability, and the potential and capacity for resource mobilization.

### Implementation theory

When people seek to implement a new way of classifying a disease, a new surgical technique, or a new way of organizing the transport of patients between hospitals, they express their agency *(i.e*., their ability to make things happen through their own actions). This is expressed in interaction with other agents, other processes, and contexts. Agents seek to make these processes and contexts plastic: for to do one thing may involve changing many others. Implementation therefore needs to be understood from the outset as a process – that is, as a continuous and interactive accomplishment – rather than as a final outcome. Moreover, ‘implementation’ never refers to a single ‘thing’ that is to be implemented. Whenever some new way of thinking, acting, or organizing is introduced into a social system of any kind, it is formed as a complex bundle – or better, an ‘ensemble’ – of material and cognitive practices. Even what appear as very simple implementation processes involve many moving parts. Throughout what follows, the term ‘complex intervention’ is therefore used to define the object of any implementation process [11-13].

**Figure 1:**
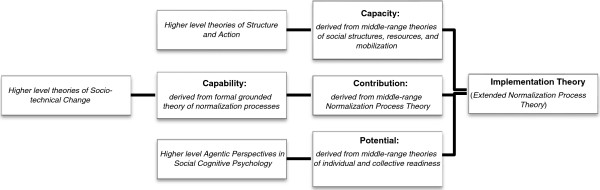
How higher level and middle-range theories are assembled to support the proposed General Theory.

The aim of implementation theory development is the production of a robust set of conceptual tools that enable researchers and practitioners to identify, describe and explain important elements of implementation processes and their outcomes. The theory presented here links together a set of constructs drawn from several theories. (These are mapped in Figure 1.) When integrated, these comprehensively describe and explain elements of a complex dynamical system.

Considerations of space mean that it is not possible to offer in this paper a comprehensive review of existing theories. (For major accounts of the problem of agency, routine and habituation, see Emirbeyer and Mische [14], Archer [15] and Camic [16], respectively. See also important papers by Grol *et al*., [7], Tabak *et al*., [17], Glasgow et al., [18] and Damschroder *et al*. [19,20], which review the bases of analytic frameworks and their application.) Other, important theory-based frameworks for implementation have also been developed using integrative techniques. In management science, the highly influential Diffusion of Service Innovations model proposed by Greenhalgh *et al*. [21], adds constructs from social psychology, organizational behavior theories, and socio-technical systems theory to produce a typology of factors that affect diffusion into practice. The Technology Acceptance Model utilized by Venkatesh *et al*. [22] also added a group of ‘diffusion’ constructs to those proposed by the Theory of Planned Behavior [23]. It appears to be predictive of intention to utilize behaviors, interventions and innovations [24]. The Theoretical Domains Framework also builds on multiple theories, combining constructs from different sources [25]. The Technology Acceptance Model and the Theoretical Domains Framework are both intra-disciplinary models that focus on individual differences and make an important contribution to understanding and evaluating change.

In the complex realm of emergent social and organizational processes of intervention and innovation, a general theory of implementation is likely to require more than an intra-disciplinary model. The range of phenomena involved means that an inter-disciplinary perspective that draws on insights from sociology and psychology is likely to offer a more comprehensive explanation of implementation processes.

### The plan of this paper

The work presented in this paper is integrative. It takes a set of already existing theoretical constructs and links them together in a new way. The first part of this work (in the introduction and the first section of the discussion) sets out some key definitions of terms that underpin the agentic approach taken here. This approach is founded on the notion that implementation expresses ‘agency*,*’ and should be understood and evaluated against the problem of how human agents take action in conditions of complexity and constraint.

In the second part of the discussion, four key elements of a general theory are laid out. These are expressions of agency within implementation processes, characterized through constructs of capability and contribution; and dynamic elements of the context of implementation, characterized through the social structural and social cognitive resources upon which agents draw when they take action – these are encompassed by constructs of capacity and potential. Each construct is described, its genealogy registered, and its core components or dimensions are defined. Each construct is also reduced to a single context-independent proposition.

**Figure 2:**
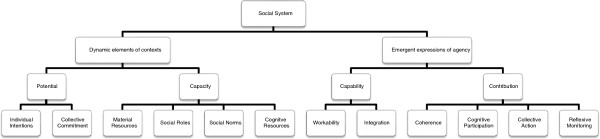
Concepts, Constructs and Dimensions of the General Theory.

**Figure 3:**
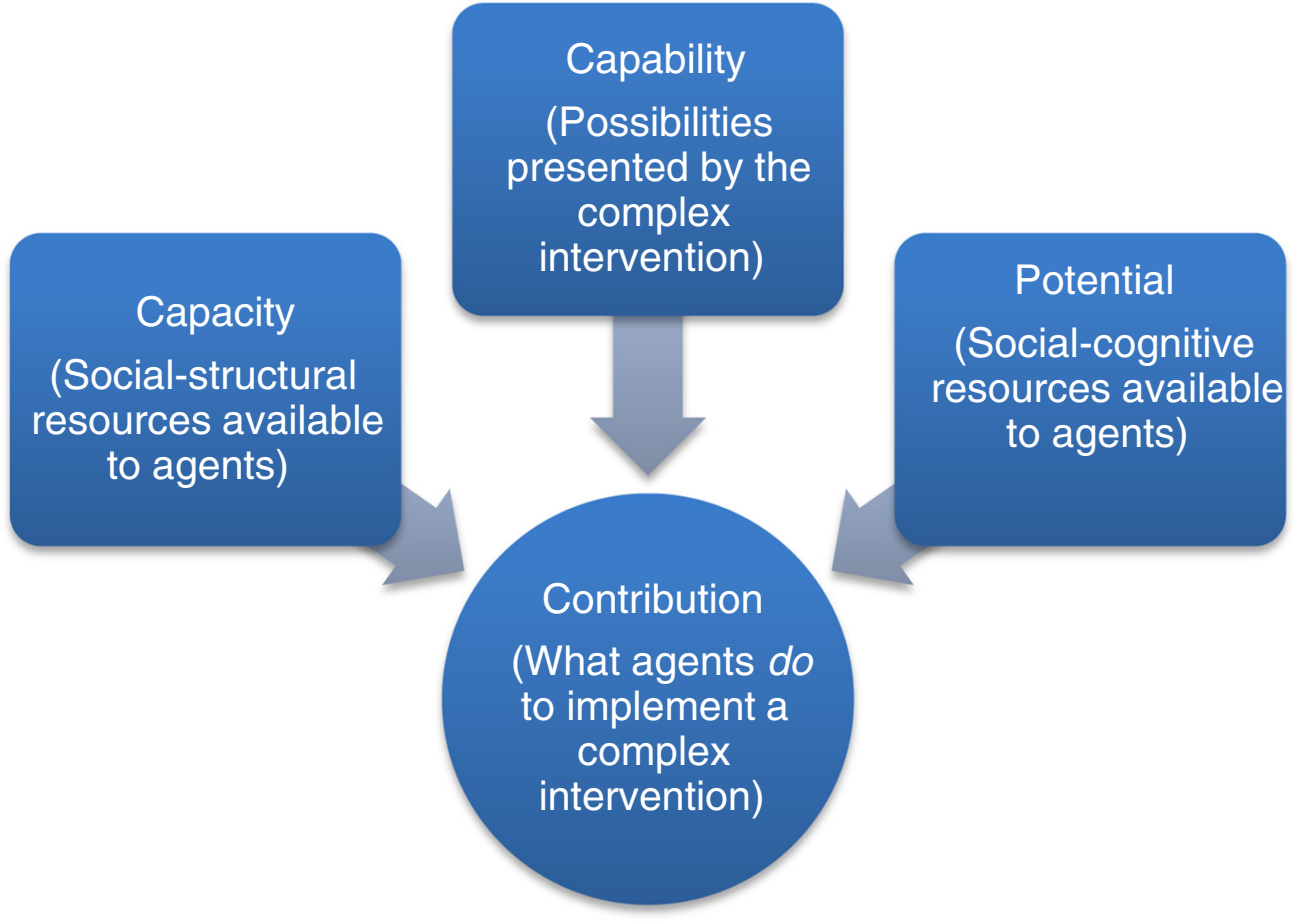
Resources and possibilities for agents’ contributions to implementation processes.

Next, the generic set of constructs and propositions that make up the proposed general theory are translated into a context-dependent narrative that characterizes elements of the implementation of clinical practice guidelines in nursing. This part of the paper also demonstrates how analytic propositions can be reassembled to form a robust low-level theory of practice. This is followed by a third section of the discussion that describes some of the limits of the theory as presented. In the summary section of the paper, additional comments are made about the relevance of the work, and a set of summary claims about the social organization of implementation processes are made. There are three figures: Figure 1 shows the ways in which higher order theories have informed the development of the constructs presented here. Figure 2 shows how the constructs of the general theory are linked, and Figure 3 shows how the concepts, constructs, and dimensions of the theory are hierarchically arranged.

### Discussion: core constructs of a general theory of implementation

The aim of implementation theory is the development of a robust set of conceptual tools that enable researchers and practitioners to identify, describe and explain important elements of implementation processes and outcomes. The proposed general theory presented here links together a set of constructs drawn from other theories. When integrated, these begin to comprehensively describe and explain elements of the processes by which implementation, embedding and integration take place. These constructs are anchored to a central theoretical claim, which is that social and cognitive processes of all kinds involve social ‘mechanisms’ that are contextualized within social systems and from which spring expressions of agency. However, before moving on to the constructs of the theory, some key terms first need to be defined.

### Definitions: system, mechanism, implementation

Before discussing the constructs of the theory, it is worth being clear about what is meant by some key terms. For the purposes of this paper, a social system is defined as a set of socially organized, dynamic and contingent relations. These relations form a structure that is populated by agents (who may be individuals or groups) that interact with each other. Information and other resources flow through these interactions between agents. As Scott notes, social processes cannot be understood without reference to social systems [26]. A system therefore forms structural conditions for the expression of agency. Social systems are emergent, which means that they are shaped, over time and across space, by both endogenous and exogenous factors. This means that their future is relatively unpredictable.

Within emergent structural conditions, social mechanisms operate. In this paper, a mechanism is defined as a ‘process that brings about or prevents some change in a concrete system’ [27], that ‘unfold[s] over time’ [28], and expresses contributions of human agency [29]. The value of a mechanism’s focused approach is that it helps us understand the means by which humans act on their circumstances and try to shape them. Here, ‘agents jointly construct their own actions as pragmatic, strategic responses to their circumstances and as expressions of commitment to their values’ [26]. In this context, a mechanism-based approach focuses on the things that agents do to make their affairs plastic or malleable.

Taken together, emergence in social systems and plasticity in social mechanisms mean that the future shape and form of any social process is uncertain. This is a view shared, for good reasons, by proponents of very different theoretical positions – from systems theory [30], to the sociology of science and technology [31]. Ideas about the importance of social mechanisms as explanations of social processes have become important as the social sciences have sought to deal with problems of contingency and causation [29,32,33].

Finally, we need a definition of implementation. For the purposes of this paper, implementation can be characterized as a deliberately initiated process, in which agents intend to bring into operation new or modified practices that are institutionally sanctioned, and are performed by themselves and other agents [34]. These act to modify a social system. As this happens, agents – who are the individuals and groups that encounter each other in healthcare settings – engage in the realization and mobilization of material and cultural resources, and secure the consent, cooperation and expertise of those other agents who inhabit the particular field or domain of action in which the process of implementation takes place [8,34-36]. Implementation subsumes all related activities from initiation to incorporation [37], and it may lead to the routine incorporation of ensembles of practice in everyday work [38,39].

### Constructs of the general theory

A theory stands or falls on the extent to which it actually illuminates and explains a set of phenomena. To perform this function it must offer a general, and context-independent, cognitive model that simplifies those phenomena. In this section of the paper, the four constructs – capability, capacity, potential and contribution – that are brought together to form the general theory are described. The relationship between these constructs is shown in Figure 2. Each of the construct descriptions outlines its theoretical antecedents, characterizes its core components or dimensions, and reduces the construct to a single context-independent proposition. The structure of concepts, constructs and dimensions is shown in Figure 3.

This section sets out the elements of the theory in the most general way, but it does not show how the theory can be operationalized in a context-dependent setting. So, in the section that follows, a worked example of the theory-in-use is presented. This applies the constructs directly to a practical problem – the implementation of nursing clinical practice guidelines – and shows how each of the theory’s general propositions can be translated into a context-dependent proposition that looks much more like a research hypothesis.

1. Capability

The first construct to be discussed is that of capability. The question of what is being implemented is always more complex than might be supposed. For the purposes of this paper, the object of an implementation process is subsumed under the ambit of a ‘complex intervention’ [11] – a cognitive and behavioral ensemble that involves different material and cognitive practices, relations and interactions. When agents engage with complex interventions, they engage with multiple objects of practice. These may include classifications, real or virtual artifacts and techniques, technologies or organizational systems. A complex intervention may include all of these, and this is an area of significant interest in the social sciences. It includes landmark studies by Burri on MRI scanners [40], and by Yoxen on the development of ultrasound [41]. New or modified ensembles of practice are often intended to change people’s expertise and actions, illustrated well in Smith *et al.’s*, study of anesthesia handovers [42]. Much work in this field has critically interrogated the development of informatics applications. See, for example, Berg’s study of decision-making tools [43], and Nicolini’s [44] and Lehoux’s [45] work on telemedicine systems. These studies have shown how the attributes of the components of complex interventions themselves affect their use. Such attributes include their virtual or physical character [46], the assumptions about use and users that are embedded within them [47,48], their complexities in practice and in the social relations that they engender [49], and their expected value. All of these elements combine to make them much more than the sum of their parts and to shape the relations between agents and the different components of a complex intervention through processes of mutual co-constitution [50-52].

The qualities of complex interventions – whether they are workable in, and can be integrated into, practice – are therefore important elements of implementation processes. In an earlier paper [38], it was shown that workability can be divided into the actual material practices that agents perform when they operationalize a complex intervention (its interactional workability), and the ways in which these practices were linked to, and distributed through, a division of labor (its skill set workability). Equally, integration can be divided into contextual integration, in which the performance of a practice is linked to the means by which it is realized and to the resources transmitted to it, and relational integration, in which the performance of a practice is linked to the means by which users make themselves and others accountable for its performance. Some existing frameworks have utilized workability constructs from diffusion of innovations theory [20,24,53], setting out, for example, ideas about ‘trialability’ and ‘ease of use’ as being important components of such models. The risk here is that these come to be seen as qualities of the objects themselves, rather than expressions of the capability of their users that are, in turn, derived from the interactions between them. Users make objects workable through use, and they work to integrate them in their social contexts.

Having explored some of the underlying theory (and empirical work) that underpins capability as a construct of the theory, the next step is to characterize its important dimensions. Here, the relational possibilities that a complex intervention presents can be defined as follows:

1.1 Workability: the social practices that agents perform when they operationalize a complex intervention within a social system, and characterizes interactions between users and components of a complex intervention;

1.2 Integration: the linkages that agents make between the social practices of a complex intervention and elements of the social system in which it is located, and characterizes interactions between the context of use and components of a complex intervention.

The object of an implementation process is some new or modified way of thinking, enacting or organizing action. An object may be virtual or concrete, or both, and it is always associated with an ensemble of cognitive and behavioral practices. It can thus be characterized as a complex intervention, and the possibilities it presents to agents can be set out in a single proposition.

**P1**. *The capability of agents to operationalize a complex intervention depends on its workability and integration within a social system.*

The implication of this is that a complex intervention is disposed to normalization into practice if its elements, and their associated cognitive and behavioral ensembles can be made workable and integrated in everyday practice by agents. If workability and integration cannot be sustained, then the embeddedness of the complex intervention will be threatened as the capacity of agents to employ it is confounded.

2. Capacity

Much work about the diffusion of innovations has started with the notion that advances in technology or practice flow through, and gradually populate, large scale social networks [54,55]. They can do this because they possess attributes that make them attractive to different kinds of ‘adopters’ [56]. Greenhalgh *et al*.’s [21] important review of diffusion of service innovations studies introduces 53 measurable attributes to this model [53]. The existence of particular kinds of social networks are important antecedent conditions for implementation processes, because they provide relational contexts for the reciprocal chains of interactions and flows of information that form social systems [57]. The mechanisms involved in flows of ideas and innovations spread are often unclear, but are assumed to be like those of mimesis or contagion [58]. However they work, networks form relational pathways through which different kinds of work are done. This means that they are accomplishments rather than static structures, and that these accomplishments include information flows and practices of operationalization of the complex intervention.

Social networks may overlay relatively ‘open systems’ that are diffuse and unbounded, and they often transcend formal institutional boundaries [59]. An example might be a population dispersed over many organizations of different sizes, and distributed in social space, like the physicians studied by Coleman *et al*., in their classic study of the diffusion of pharmaceutical products [60]. Or, they may overlay relatively ‘closed systems’ that appear to be highly structured and bounded. These may be specific organizations, or work groups, like those discussed by Whitten in her work on the diffusion of telemedicine services [61,62]. They may also take the form of highly structured and bounded networks that exist within – or between – organizations. An interesting example is that of the networks involved in designing, delivering and participating in large randomized controlled clinical trials [63]. These can be complex and widely distributed (often internationally) but remain highly structured and have robust mechanisms to ensure their closure.

The value of social network theories to understanding the dynamics of implementation processes is that they enable the characterization of the relational pathways between agents (and groups of agents), and explanation of their effects. Strategic Action Field Theory [36,64] has the potential to facilitate understanding of implementation dynamics from a different standpoint, which is the analysis of the field in which an implementation process occurs. This may be a macro-level field (in the case of large-scale policy implementation across a whole healthcare system), a meso-level field (in the case of organizations or clusters of organizations that form a sub-set of a large-scale implementation program), or micro-level fields (in the case of specific workplaces, teams, families, or other small groups). Many implementation processes encompass activities within all of these domains, with fields being ‘nested’ within each other, being arranged in vertical hierarchies, or horizontally overlapping each other. However it is situated, a field is defined as a ‘fundamental unit’ for collective action that takes the form of a ‘social order where actors (who can be individual or collective) interact with knowledge of one another under a set of common understandings about the purposes of the field, the relationships in the field (including who has power and why), and the field’s rules’ [36]. Within such fields, agents work together in skilled ways to achieve goals and facilitate the engagement and co-operation of others.

The ability to engage others in collective action is a social skill that proves pivotal to the construction and reproduction of local social orders (…) Social life revolves around getting collective action, and this requires that participants in that action be induced to cooperate. Sometimes coercion and sanctions are used to constrain others. But often, skilled strategic actors provide identities and cultural frames to motivate others [64].

This kind of theoretical perspective enables the analysis of basic conditions for the expression of agency that participants invest in implementation. They exercise their capacity to do this in fields that may be hierarchically nested and, or, overlapping and that provide interactional structures for the variable distribution of people, power and resources. Within these bounds, participants are characterized by a variety of context-dependent affiliations, social roles, and rules in the form of social norms and conventions. These may include the capability to define and regulate conduct by consensual or coercive means [65].

The problem of the capacity of a social system to accommodate an implementation process is bound up with the extent to which it offers a set of social-structural resources to the agents that inhabit it. Once again, we can define important dynamic elements of the context of implementation as a set of dimensions of the construct, thus:

2.1 Social norms: institutionally sanctioned rules that give structure to meanings and relations within a social system, and that govern agents’ membership, behavior and rewards within it. They frame rules of membership and participation in a complex intervention.

2.2 Social roles: socially patterned identities that are assumed by agents within a social system, and that frame interactions and modes of behavior. They define expectations of participants in a complex intervention.

2.3 Material resources: symbolic and actual currencies, artifacts, physical systems, environments that reside within in a social system, and that are institutionally sanctioned, distributed and allocated to agents. They frame participants’ access to those material resources needed to operationalize the complex intervention.

2.4 Cognitive resources: personal and interpersonal sensations and knowledge, information and evidence, real and virtual objects that reside in a social system, and that are institutionally sanctioned, distributed and allocated to agents. They frame participants’ access to knowledge and information needed to operationalize the complex intervention.

Implementation of a complex intervention occurs when agents deliberately attempt to initiate its incorporation within a social system, in a way that modifies the operation of that system and changes its possible outcomes. It thus affects the social roles, norms and conventions that govern the conduct of agents [66,67], and the material and informational resources available to them, within a set of dynamic and contingent interactions. This can be expressed through a single proposition.

**P2***. The incorporation of a complex intervention within a social system depends on agents’ capacity to cooperate and coordinate their actions*.

The implication of this is that a complex intervention is disposed to normalization into practice if the social system in which it is located is one that provides normative and relational capacity – through which agents resource, cooperate, and coordinate their investments and contributions to its use. If capability cannot be sustained, then the embeddedness of the complex intervention will be threatened as its context of action decomposes.

3. Potential

Social systems theories of different kinds are important foundations for analyses of implementation processes because they enable us to characterize the normative structures in which roles, rules and resources reside, and through which they are distributed. Ideas about fields, structured interaction processes and relations, and the mechanisms of control and network transmission that they make possible, therefore set out important conditions for implementation processes. They characterize important relational features of the dynamic social contexts in which agents are situated. But the presence of fields, social networks and interaction chains, and mechanisms for their regulation and control are important but insufficient to understand the dynamics of implementation. Here, potential agency [14] and motivation [68] are themselves necessary antecedents for the dynamic and emergent conditions that follow. In this context, agency is a quality that can be characterized as:

a temporally embedded process of social engagement, informed by the past (in its habitual aspect), but also oriented toward the future (as a capacity to imagine alternative possibilities) and toward the present (as a capacity to contextualize past habits and future projects within the contingencies of the moment) [14].

Psychological theories play an important part in conceptualizing the ways in which potential is an antecedent condition for implementation, and is linked to agency [23,69-73]. The construct of *potential* defines a starting point for understanding the antecedent conditions for implementation processes. To make the best of these theories, we can see them as focusing on individual [23], and collective [71], commitments. Individual intention is an antecedent condition for action that is especially important in circumstances where it can be shown that agents possess significant degrees of professional autonomy or personal discretion to pursue their interests [74]. But, in the context of potential as a property of individual members of a social system, it makes more sense to think about collective processes. The construct of organizational readiness is valuable here, and Weiner [71] sets out a highly relevant theoretical model that rests on two concepts, change *valence* and change *efficacy*. The first of these is characterized as the degree to which organizational members collectively value the change that an implementation process will bring about. Weiner argues that if they value it enough, then they will commit to it. The second, is characterized as ‘a function of organizational members' cognitive appraisal of three determinants of implementation capability: task demands, resource availability, and situational factors’ [71]. An important feature of Weiner’s approach is that it.

treats organizational readiness as a shared team property – that is, a shared psychological state in which organizational members feel committed to implementing an organizational change and confident in their collective abilities to do so. (…) Some of the most promising organizational changes in healthcare delivery require collective, coordinated behavior change by many organizational members [71].

Weiner sets out a highly interactive model in which important features of context, such as organizational culture and operational environment, are expressed through change valence and change efficacy. It is highly interactive, too, in the sense that it emphasizes the accomplishments, shared values and commitments of groups. No matter how much individual potential and commitments are valued socially, implementation processes are largely collective and collaborative in their form and direction. We can clearly define two translational mechanisms at work here, and these form the key dimensions of the construct.

3.1 Individual intentions*:* agents’ readiness to translate individual beliefs and attitudes into behaviors that are congruent, or not congruent, with system norms and roles. They frame individual motivation to participate in a complex intervention.

3.2 Shared commitments*:* agents’ readiness to translate shared beliefs and attitudes into behaviors that are congruent, or not congruent, with system norms and roles. They frame shared commitment of participation in a complex intervention.

Realizing agents’ capability to implement a complex intervention into action to achieve their goals depends on them being disposed to do so. These dispositions are expressed through individual attitudes and intentions, and shared values and commitments. These may depend on agents’ beliefs about attributes of the complex intervention and their beliefs and experiences of capability. They can be expressed as a single proposition.

**P3**. *The translation of capacity into collective action depends on agents’ potential to enact the complex intervention.*

The implication of this is that a complex intervention is disposed to normalization into practice if agents both individually intend and collectively share a commitment to operationalizing it in practice. If potential cannot be sustained, then the embeddedness of the complex intervention will be threatened as agents’ commitments are withdrawn.

4. Contribution

So far, it has been seen that social systems are formed when social roles and norms are accomplished with organized, dynamic and contingent patterns of interactions. These may be described through theories of social networks and characterized through dynamic field theories. Within the fields thus characterized, populations of agents (whether these are individuals or groups) interact with each other, and information flows between them. As this happens, individual intentions and collective commitments are formed and expressed. We thus have a theoretical vocabulary for characterizing both the social environment of, and agentic potential for, implementation in a generic or context-independent way. Here, as Bandura puts it, being an agent is about enacting intentionality and potential.

To be an agent is to intentionally make things happen by one’s actions. Agency embodies the endowments, belief systems, self-regulatory capabilities and distributed structures and functions through which personal influence is exercised, rather than residing as a discrete entity in a particular place. The core features of agency enable people to play a part in their self-development, adaptation, and self-renewal with changing times [73].

This leads us to the next point to consider. This is an important theme in recent theory development about implementation-as-action. Here, May and Finch [8], Weiner [71], Colyvas and Jonsson [35], and Fligstein and McAdam [36], have all – from very different theoretical perspectives – pointed to the importance of analyzing elements of change from the perspective of, as Weiner [71] calls it, ‘collective, coordinated, and co-operative social action.’ This problem of collective, coordinated and cooperative social action is the pivot upon which implementation – and thus implementation theory – must turn. In this context, Normalization Process Theory [8] is one of a number of theories – including Activity Theory [75], Labor Process Theory [76], Structuration Theory [77], and Neo-Institutionalist Theory [78,79] – that can be applied to understand agents at work within implementation processes. In psychological theories of agency, like those proposed by Bandura [72], it is individuals that matter. But agency need not be considered a property of individuals alone.

[F]orms of joint action can unite two or more individuals towards a shared end. In joint action, disparate individuals are coordinated in such a way that they become centered on each other (…) and are able to act collectively, as if they were a single entity. In certain circumstances, then, complex structures of jointly acting individual agents are able to act as collectivities [26].

Joint action of this kind expresses the operation of social mechanisms that are characterized by Normalization Process Theory [8,10]. These generative mechanisms are visible when agents’ contributions in collective action lead to the definition and meeting of goals, and their operation is shaped by organizing structures and social norms [66]. These specify the rules and roles that frame action, and the group processes and interactional conventions [80] through which action is accomplished. Once again, we can develop a more detailed picture of these mechanisms and characterize them as a set of dimensions.

4.1 Coherence or Sense-Making: agents attribute meaning to a complex intervention and make sense of its possibilities within their field of agency. They frame how participants make sense of, and specify, their involvement in a complex intervention.

4.2 Cognitive Participation: agents legitimize and enroll themselves and others into a complex intervention. They frame how participants become members of a specific community of practice.

4.3 Collective Action: agents mobilize skills and resources and enact a complex intervention. They frame how participants realize and perform the intervention in practice.

4.4 Reflexive Monitoring: agents assemble and appraise information about the effects of a complex intervention within their field of agency, and utilize that knowledge to reconfigure social relations and action. They frame how participants collect and utilize information about the effects of the intervention.

When agents enact a complex intervention, they collectively express the operation of social mechanisms. Through these, they make contributions in dynamic reflexivity, continuously making and acting upon their sense of the form and application of a complex intervention, at the same time appraising its effects. Equally, they invest in directed action, continuously building and acting upon the relational features, and performing the material practices needed to implement and embed the complex intervention in practice. This leads us to a final proposition, drawn directly from earlier work [8]. It is that:

**P4**. *The implementation of a complex intervention depends on agents’ continuous contributions that carry forward in time and space.*

The implication of this is that a complex intervention is disposed to normalization into practice if agents invest in operationalizing it in practice. If contribution cannot be sustained, then the embeddedness of the complex intervention will be threatened as agents’ efforts diminish.

### Application of the theory: a worked example

In the preceding section, the general theory was presented as a set of context-independent constructs, dimensions and propositions. The question that arises from this is, how would we use this general theory to structure understanding of an implementation process? This is as much a methodological question as it is a theoretical one, but it is important to illustrate the theory in action. In this section of the paper, the context-independent constructs and propositions of the theory are translated into the context-dependent form of a worked example.

The worked example will be presented in two stages. First, a theory-informed narrative of the implementation of a new clinical practice guideline for nurses will be presented. Second, the context-independent propositions of the general theory will be translated into context-dependent ones, to provide a specific theoretical framework for planning and evaluating the implementation of clinical practice guidelines.

It must be emphasized that this is a worked example of a theory in practice, not a formal data analysis or review, but it does draw on information from seven studies [81-87] that have met the quality criteria for inclusion in a systematic review of qualitative studies of nursing guideline implementation informed by Normalization Process Theory.

### Implementation of clinical practice guidelines in hospital nursing: theoretical narrative

The starting point for the worked example is to consider the dynamic features of context in which an implementation process takes place. Here, the implementation of a clinical practice guideline is an intentional modification of the existing routinely embedded relationships and practices through which the hospital department is constituted a social system. These are already highly structured, with formal and informal norms that govern the conduct of work by nurses and other professionals, and well-defined professional roles that they assume when they do so. At the same time, nurses working in this setting have available to them a body of cognitive and material resources that provide the basis of knowledge and practice for their work. These social-structural resources make being a nurse and doing nursing work possible. The introduction of the guideline changes to some extent their organization and allocation. By definition, it changes the rules or norms that govern the conduct of work and, if it involves the re-allocation of work from one group of professionals to another, it may also change their roles. Introducing the guideline may also change the distribution and availability of material and cognitive resources available to nurses and other professionals.

In circumstances where nurses did not cooperate with each other over changing norms or roles, or resisted the coordination of changes in material and cognitive resources, we might expect the prospects for normalization of the guideline to diminish. There is of course a second dynamic feature of context, which is the potential of nurses to engage with the work of operationalizing the changes that implementing the guideline brings with it. In this context, the attitudes and intentions of individual nurses (especially in situations where they have a high level of personal autonomy) are important. These play into a wider set of shared commitments, in which nurses build a sense of collective readiness, not simply to enact the guideline but also to work to accommodate the other changes that it will bring. In this context, collective readiness is interdependent with, but not simply the sum of, individual attitudes. As Weiner points out [71], shared commitments is a complex phenomenon, but plainly this is also highly relevant to the problem of capacity. The relationship between potential and capacity is a complex one, since nurses’ understandings of what must change during the implementation of a guideline are likely to shape readiness to act. Certainly within social systems of all kinds, dynamic elements of contexts such as those specified by notions of capacity and potential shape each other. But they also continuously interact with emergent expressions of agency as a social process is formed.

Turning now to emergent expressions of agency, we can begin by thinking about how nurses work upon a clinical guideline. A clinical practice guideline is a set of procedures that are intended to govern practice, and which are embedded in software (perhaps in an electronic healthcare record, or some other system) or in hardware (in a bedside card, paper record, or printed set of standard operating procedures). It will embody a set of assumptions about the context in which it is to be used, and about the nature of the user, which will in turn shape its relationship with that context and structure the way that it is practically used. So, rather than seeing the guideline as a ‘thing’ to be implemented, it is better understood as a set of practices. These have varying degrees of workability (the ways in which they can be deployed and acted upon by their users) and integration (the ways in which they express expectations of their users and conditions of use). These assumptions and expectations may not be correct – indeed, a common experience of implementation of complex interventions of all kinds is that they need to be locally reinterpreted and modified in practice – and the use of a guideline may have unanticipated consequences, even if it is deployed as intended.

Finally, while nurses are able to draw upon and mobilize social-structural and social-cognitive resources and potential as they proceed through the implementation of a clinical guideline, and while their capability to do so is related to its workability and integration, it is the actual doing of the guideline in practice that matters. This is important because there are ample examples of the implementation of complex interventions where individual and shared commitment to implementation is revealed to be low, and where the social and cognitive resources available to nurses are massively disrupted, and yet professionals are able to reconfigure practice to make it ‘work’ – and *vice versa*. So it is what nurses actually do when they implement a clinical practice guideline that must be at the center of analysis.

The basic claim of the theory [8] is that the course of an implementation process is governed by the operation of social mechanisms that are energized and operationalized through agents’ contributions. In this case, it means that nurses work to make sense of the guideline and work out how to put it into action. In this context, they need to think through what the guideline will mean for practice (and how it will make practice different). This sense-making work may be quite informal, but it fulfills an important function, which is to make the body of everyday work into a coherent whole and to give it a sense of orderliness. At the same time, all of the participants in the implementation of the guideline – who may also include patients, their significant others, and other professionals and administrators – also need to find ways to bring about a community or practice in which the guideline is seen as initiating and enrolling them into a legitimate reconfiguration of practice. These are important antecedents for ‘doing’ the guideline in practice because they form points of connection between nursing work and its structural and cognitive resources, but they are also continuing accomplishments as the guideline is enacted in everyday practice.

It is collective action – nurses working together to put the guideline into practice and continually using it with their patients (or not) that is the central element of the implementation process. For it is here that the guideline ultimately becomes normalized and disappears from view as it becomes the ‘way we do things here.’ As this collective action continues, so too does the work of appraisal – which may be some formal evaluation of the guideline, but is almost certainly also an informal collection of experiential accounts and implicit theories about why things turn out as they do. The theory depends on this notion of agentic contributions (and the investments in agency through which they are formed). It is that agents (who may be individuals and groups) mobilize resources (which may be both structural and cognitive) and then invest them in enacting the ensemble of practices that make up the work of implementation.

### Implementation of clinical practice guidelines: context-dependent propositions

Focusing on the implementation of clinical practice guidelines in nursing is interesting. They are hard to implement. Implementation and embedding in practice take place in complex organizational and clinical environments, in circumstances where time is both a scarce personal asset and an expensive corporate asset, and where work of one kind is constantly squeezed by other demands. This forms the background of a theoretical narrative that accounts for implementation – in the wider contexts of multiple sources of contingency and a wide variety of confounding factors – the next step is to take that theoretical narrative and translate the theory’s propositions into a context-dependent form. Taking this step is important because the purpose of the theory is to help facilitate both prospective understanding of implementation processes and evaluation of their outcomes.

First of all, we can consider the two dynamic elements of context that the theory specifies. These provide social and cognitive resources on which agents (in this case nurses and their associated professionals) draw when they work to negotiate the complex working environment in which they are set, and implement the guideline.

**Capacity**: *The implementation of a clinical guideline in its practice setting depends on nurses’ capacity to: (i) cooperate to operationalize changing norms and roles; and (ii) coordinate their operationalization of changing material and cognitive resources*.

**Potential**: *The translation of nurses’ capacity into contributions to practice change depends on the degree of: (i) their individual intentions; and (ii) their shared commitments to enact the guideline.*

No claims are made here about the relations between capacity and potential. Whether one is contingent on the other is a matter that must be determined empirically. The next step is to consider the two emergent expressions of agency that the theory specifies. These focus on the agentic relations between nurses and the guideline, and the work that nurses do to incorporate the guideline into their workstream.

**Capability**: *The capability of nurses to implement and embed a clinical guideline in everyday practice depends on its qualities of: (i) workability at the bedside; and (ii) integration within nurses’ workflow.*

**Contribution**: *The implementation of a clinical practice guideline depends on nurses’ continuous contributions of agency to: (i) continuously enact it; and (ii) carry it forward as an element of future work.*

Once again, the contingent relations between these two constructs (and their relations with dynamic elements of their context) must be determined empirically. For each of these, we now have a pair of context-dependent propositions. These can be worked up as specific hypotheses for a prospective study of guideline implementation, but at the moment they function as a low-level theory. Once again, this is important: translational theories such as this one provide a realistic degree of granularity, both for planning an implementation process, and evaluating its progress and outcomes.

### Limitations of the theory

Thus far, the possible constructs of a general theory have been outlined; key components of these constructs have been identified and defined; and a set of propositions have been laid out. The first of these characterize domains in which social mechanisms operate, the second characterize specific foci of empirical investigation and measurement, and the third provide the foundations for a set of testable hypotheses about the course and direction of implementation processes themselves. These can be combined with those set out in two earlier papers [8,29] to provide a more comprehensive explanatory model of processes of implementation, embedding and integration of complex interventions**.**

The description of constructs, thus far, shows a set of mechanisms that energize and shape implementation processes. It also suggests how endogenous factors might confound these processes, for example through the withdrawal of agents’ shared commitment to a complex intervention, or through some failure of workability and integration. Plainly, there are many reasons why implementation processes take the form that they do. Many of them involve exogenous factors. Fligstein and McAdam [36] call these ‘shocks,’ and they also include what proponents of actor-network theory call ‘contingencies’ [1], which arise outside of the fields in which the implementation process takes place. Their effect is best determined empirically: there is no need to account for every possible permutation of contingency and confounding. We know for example that wars; epidemics; financial crises; changes of government, law and policy; organizational strategizing, collapse or takeover; resistance and recalcitrance on the part of other systems of practice; and the emergence of other new techniques and technologies all have such effects. However, in such circumstances, agents often continue to invest in overcoming turbulence and recalcitrance, and seek to make their effects malleable and plastic.

Limits must be placed on integrative theories such as this one. First, psychological and sociological theories that have been drawn on here variously place individual cognition and agency at their centers, while others give primacy to social processes. For the moment, we have to put this problem to one side; the debate about the relationship between structure and agency is a meta-theoretical problem. At a more practical level, although a comprehensive theoretical model of implementation processes would be a valuable tool for practitioners and researchers, the phenomena that are involved are so numerous, variable and complex that it may be that they cannot be fully captured. In relation to this, it is important to note that comprehensiveness and omniscience are not the same thing, just as federation and unification are also different. The aim in this paper is to move towards a general theory by producing a more comprehensive model, not by enumerating all phenomena and unifying all possible theories.

Finally, while sensitivity to theory and awareness of its diverse forms and purposes is a normal part of the training of social scientists, the integration of constructs belonging to different theories is an under-explored problem of method [88]. There is no universally accepted technique for accomplishing this task. These limits aside, the strength of the analysis offered here lies in its middle-range operationalization and the modest claims that are consequent to this.

### Summary

At a time when most healthcare systems are under tremendous pressure, why should we be concerned with theory? Surely there are enough theories, and there are enterprises that are more practically useful to policy-makers, clinicians and researchers? The justification for doing such work is, in this context, a simple one. There is much evidence about the clinical and cost-effectiveness of new and existing treatment modalities, and ways of delivering and organizing care. What ‘works’ is – in many fields – established through rigorously designed and applied outcomes studies. But it is far less clear, to clinicians in practice as well as to policy-makers and managers, how to get these advances in healthcare and its delivery into practice, and – on that implementation journey – how to understand the factors that will promote or inhibit their passage. Robust theories form the foundation for rigorous research to inform implementation journeys [17].

### Theory-building as a journey

The claim of a general theory is one that invites hubris, and the claim that this work is on a journey towards a general theory only reduces this prospect a little. However, implementation science is a field where interest in developing and testing new theories and theory-informed evaluation and planning frameworks is exploding. This makes the field intellectually exciting and practically interesting. It is against this background that the proposed General Theory has developed.

As Figure 1 suggests, the theory presented here is a waypoint on another kind of continuing journey, too. This is a theoretical journey that began with the development of a formal grounded theory (the Normalization Process Model [34,38]) that explained aspects of the routine incorporation of complex healthcare interventions into practice. This model was then developed into Normalization Process Theory, a generic and middle-range theory of implementation [8,10]. In the present paper, the theory has been further extended. Integrated with constructs from other theories, a more comprehensive set of explanations for implementation processes is formed. Integration has included constructs related to the structural properties of social systems, and individual and shared intention, to those related to the attributes of complex interventions and to the collective action of their users. The approach taken throughout has been to sketch out social processes and relationships and their associated mental and social mechanisms. In this context, including perspectives from higher level accounts of socio-technical change [50], agentic perspectives in social cognitive psychology [73], and social theories of structure and action [89] – permits more comprehensive explanation.

The four constructs derived from this work – capacity, potential, capability and contribution – define the core of a parsimonious and workable general theory of implementation based on social mechanisms. The relationships between them are mapped in Figure 3. They have regard for the dynamic elements of the contexts and objects of implementation, and for the dynamic potential and actual expressions of agency. These form the social processes through which implementation is accomplished. They are not linear or sequential, but interact continuously with each other in emergent and complex ways. Agents’ experiences of these processes vary across social time and space, as they are shaped, encouraged and confounded by other endogenous and exogenous factors. Importantly, these constructs and their relationships with each other are not resistant to formalization. The propositions that are associated with them open this up. They represent properties of implementation processes that are multidimensional and multifactorial, but which are amenable to empirical investigation and measurement [90]. These properties are summarized in Figure 2, which sets out the hierarchy of constructs of the theory linking each level to the problem of organizing the complexity beneath.

### How implementation processes can be understood

Developed and extended in the ways that have been described in this paper, the theory asserts that implementation processes should be understood in the following terms:

1. An implementation process involves agents in the intentional modification of the social systems that occupy a field, or fields, of action.

2. Within social systems, emergent expressions of agency both shape, and are shaped by, dynamic elements of their contexts. They continuously interact to form an emergent social process.

3. Emergent expressions of agency and dynamic elements of context continuously interact with both endogenous and exogenous contingencies and confounders.

4. Agents work to negotiate the effects of interactions, contingencies, and confounders. They seek to make these plastic and shape them through their agentic contributions, and thus to govern the conduct of an implementation process and its outcomes.

Each of these characteristics of an implementation process also corresponds to a ‘level’ of analysis in the hierarchy of constructs shown in Figure 2.

In the work that has led to this paper, only constructs that characterize social or cognitive mechanisms associated with agency, and that are linked to empirical research, have been utilized. The constructs offered here are ones that can be traced back to rigorous studies that have robustly investigated processes, relations and mechanisms that have actually been shown to matter in studies of implementation and its related phenomena. The theory thus characterizes implementation processes from a position of strength. It provides a framework for thinking and planning the implementation of complex interventions, as well as a point of departure for measuring and evaluating progress and outcomes. Such interventions are to be found everywhere. They exist not just in healthcare but also in government, business, and military operations.

## Competing interests

CRM is a member of the editorial advisory board of *Implementation Science*.

## Authors’ contributions

CRM is the sole author of the paper.
